# Innovative Approaches to Safe Surgery: A Narrative Synthesis of Best Practices

**DOI:** 10.7759/cureus.49723

**Published:** 2023-11-30

**Authors:** Amer Kamal Hussain, Muhammad Maaz Kakakhel, Muhammad Farhan Ashraf, Muhammad Shahab, Fahad Ahmad, Faizan Luqman, Mahmood Ahmad, Ayman Mohammed Nour, Giustino Varrassi, Satish Kinger

**Affiliations:** 1 Urology, Sandwell and West Birmingham Hospitals National Health Service (NHS) Trust, Birmingham, GBR; 2 Trauma and Orthopaedics, Liverpool University Hospitals National Health Service (NHS) Foundation Trust, Liverpool, GBR; 3 Trauma and Orthopaedics, Wythenshawe Hospital, Manchester, GBR; 4 Trauma and Orthopaedics, Our Lady's Hospital, Navan, IRL; 5 Upper Gastrointestinal Surgery, University Hospitals Birmingham National Health Service (NHS) Foundation Trust, Birmingham, GBR; 6 Ophthalmology, Khyber Medical College, Peshawar, PAK; 7 Ophthalmology, Medical Teaching Institution (MTI) Khyber Teaching Hospital, Peshawar, PAK; 8 Trauma and Orthopaedics, Royal College of Surgeons, Dublin, IRL; 9 Pain Medicine, Paolo Procacci Foundation, Rome, ITA; 10 Medicine, Civil Hospital Karachi, Karachi, PAK

**Keywords:** surgeons, artificial intelligence, robotics, surgery, surgical safety

## Abstract

By encompassing a wide range of best practices within the ever-changing realm of modern surgical care, this exhaustive narrative compendium attempts to unravel the complex tapestry of novel approaches to safe surgery. Within the context of a dynamic surgical environment, this research endeavors to illuminate and integrate state-of-the-art methods that collectively methodically improve patient safety. The narrative elucidates a diverse array of practices that seek to revolutionize the paradigm of safe surgery, emphasizing technological progress, patient-centric approaches, and global viewpoints. The combined effectiveness of these methods in fostering an all-encompassing culture of safety, improving surgical precision, and decreasing complications is revealed by the results obtained from their implementation. The recognition of the dynamic interplay among multiple components, including the active participation of patients, the integration of cutting-edge technologies, and the establishment of comprehensive quality improvement programs, is fundamental to this narrative. By their collective composition, these components support the notion that secure surgical practices are intricate and interrelated. The present synthesis functions as a fundamental resource for healthcare professionals, policymakers, and researchers, providing an enlightening examination of the current condition of secure surgical practices. By emphasizing the promotion of innovation, continuous development, and the utmost quality of patient care, it offers a strategic guide for navigating the complex terrain of safe surgery. In the ever-evolving landscape of surgical care, this narrative synthesis serves as a guiding principle for stakeholders striving to understand better and implement safe surgical procedures in various healthcare environments.

## Introduction and background

Patient safety is at the core of every healthcare endeavor and nowhere is it more crucial than in the realm of surgical procedures. The intricate nature of surgeries, coupled with the inherent risks, demands a meticulous focus on ensuring the well-being of the patient throughout the entire surgical journey [1]. As medical science advances and surgical techniques become more sophisticated, the landscape of surgical safety is evolving, prompting the exploration and implementation of innovative approaches to enhance patient outcomes. The significance of patient safety in surgical procedures cannot be overstated. Surgical interventions, while often life-saving, inherently carry risks ranging from infection to more severe complications [2]. Ensuring patient safety is not merely about preventing adverse events; it is about optimizing every aspect of the surgical process to minimize risks and enhance overall outcomes. Patients trust healthcare providers with their lives during surgery, and the responsibility to uphold that trust rests on the commitment to the highest standards of safety [3].

Patient safety in surgery encompasses a broad spectrum of considerations, including preoperative assessment, intraoperative care, and postoperative management. Each phase presents unique challenges, necessitating a comprehensive and multidisciplinary approach [4]. From the initial screening of patients for surgical candidacy to the final stages of postoperative recovery, a focus on safety permeates every decision and action. The landscape of surgical safety is dynamic, marked by continuous advancements in medical knowledge, technology, and procedural techniques. Historically, surgical safety has primarily been associated with a reduction in immediate perioperative complications [5]. However, contemporary perspectives emphasize a broader, more holistic approach, considering long-term outcomes, quality of life, and patient satisfaction [6].

The evolving landscape of surgical safety is characterized by a paradigm shift from a reactive to a proactive model. Traditionally, safety measures were often implemented in response to identified risks or adverse events. Modern surgical safety initiatives, however, advocate for a proactive stance, integrating preemptive strategies to identify and mitigate potential hazards before they escalate [7]. This shift is in line with the broader trend in healthcare toward a patient-centered, value-based approach. As the understanding of surgical safety deepens and the demands for improved patient outcomes increase, there is a pressing need for innovative approaches. The traditional methods that have served the medical community well must be augmented by novel strategies that leverage technological advancements, data analytics, and interdisciplinary collaboration [6].

Innovation in surgical safety is not merely about introducing new gadgets or tools; it is about reimagining the entire surgical process. From the moment a patient is identified as a candidate for surgery to the postoperative follow-up, innovative approaches seek to optimize every step [8]. This might involve the integration of artificial intelligence (AI) in preoperative risk assessment, the use of advanced imaging techniques for precision in surgery, or the implementation of telemedicine for postoperative care. While the call for innovative approaches is clear, it is essential to acknowledge the challenges inherent in implementing change within the complex healthcare system. Resistance to change, financial constraints, and the need for comprehensive training are potential obstacles that must be navigated [9]. However, these challenges present opportunities for collaboration among healthcare professionals, researchers, policymakers, and technology developers to create solutions that are both effective and sustainable [10].

The importance of patient safety in surgical procedures is paramount, and the evolving landscape of surgical safety demands a proactive and innovative approach. As we delve into the narrative synthesis of best practices, it becomes evident that the challenges are as significant as the opportunities. Through a careful exploration of these challenges and opportunities, we can unravel the potential of innovative approaches to reshape the future of safe surgery.

## Review

Historical perspective on surgical safety

The historical progress of surgical safety is a story of continuous development, reflecting the increasing comprehension of the complexities involved in medical procedures. During ancient times, surgical procedures were performed in rudimentary settings, requiring essential knowledge and tools to ensure patient safety [1]. The advent of anesthesia in the mid-19th century brought about a significant change, as it revolutionized the field of surgery by effectively relieving patients from the intense agony they experienced. Following that, the latter part of the 19th century experienced a significant change in thinking with the promotion of antiseptic methods by Ignaz Semmelweis and Joseph Lister, leading to a period of emphasis on controlling infections [2]. In the early 20th century, aseptic procedures were adopted, creating sterile surgical conditions and reducing the chances of contamination. The 20th century witnessed ongoing progress in anesthetics, reducing risks connected with their administration. In the later part of the 20th century, there was a significant increase in technological advancements [3]. These advancements included the introduction of diagnostic imaging, minimally invasive surgery, and using lasers, which enhanced precision. However, they also brought up new issues requiring continuous surgical safety protocol adjustments [4].

Surgical safety in the present-day environment encounters certain obstacles that require ongoing adjustment and enhancement. The escalating intricacy of surgical treatments, including cutting-edge methodologies like robotic surgery and laparoscopy, presents difficulties in ensuring that surgical teams have sufficient training to traverse these sophisticated interventions proficiently [5]. Antibiotic resistance, an escalating worldwide issue, presents a substantial risk to managing infections in surgical environments, necessitating strategic methods for antibiotic utilization and strict adherence to sterile procedures. The increasing number of elderly individuals, who often have many simultaneous medical conditions, makes preoperative decision-making more complex [6]. This requires tailored strategies to reduce risks. Efficient communication and coordination among surgical teams are crucial for ensuring patient safety. Standardized standards, such as the World Health Organization (WHO) Surgical Safety Checklist, significantly minimize errors. Issues arising from breaks in communication, intricate procedures, antibiotic resistance, and many medical conditions in patients underscore the need for enhancement [7]. Enhancing communication, continuous training, prudent antibiotic utilization, and personalized preoperative evaluations collectively advance surgical safety [8]. The healthcare community's dedication to maintaining the highest standards of patient care is seen in its ability to adapt and overcome past milestones and current problems in surgical safety protocols.

Technological innovations in surgical safety

Technological progress has been crucial in changing the field of surgical safety, providing creative solutions that increase accuracy, decrease invasiveness, and boost patient results. A significant advancement is the incorporation of robotics into surgical procedures [8]. Robotic-assisted surgery offers exceptional accuracy, as robotic devices grant physicians improved agility and three-dimensional visualization. This allows for more complex surgeries and reduces damage to nearby tissues, resulting in quicker recovery times and fewer difficulties after surgery. Moreover, AI has emerged as a potent instrument in preoperative planning, risk evaluation, and intraoperative decision-making. AI systems can examine extensive datasets to forecast future difficulties, enabling surgeons to customize their strategy for each unique patient [9]. Advanced imaging modalities, such as magnetic resonance imaging (MRI) and computed tomography (CT), offer surgeons intricate anatomical data, enabling precise planning and execution of medical procedures. These technological advancements collectively lead to a fundamental change in surgical safety, highlighting accuracy, effectiveness, and individualized treatment [10].

Human Factors and Communication

Although there is no denying the significant impact of technical advancements on surgical procedures, human involvement remains crucial in ensuring surgical safety. The influence of human factors on patient outcomes is of utmost significance. Efficient communication, collaboration, and effective operating room leadership are essential in establishing a secure surgical setting [11]. The intricacy of surgical procedures necessitates flawless communication among all team members, ranging from surgeons to nurses to anesthesiologists. Standardized standards, such as the WHO Surgical Safety Checklist, are vital in promoting effective communication and minimizing the likelihood of errors. Furthermore, the significance of collaboration must be considered. An effectively synchronized surgical team improves productivity, diminishes the probability of errors, and cultivates a culture of cooperation. Effective leadership in the operating room is crucial, as a competent and confident leader directs the team through every stage of the procedure [12]. Human factors, including stress, weariness, and situational awareness, can influence decision-making and overall performance. Hence, it is crucial to prioritize enhancing surgical safety by effectively addressing these variables through appropriate training, consistent team building, and a dedicated focus on the human dynamics within the operating room [13].

Robotics Integration

Incorporating robotics into surgical operations signifies a pioneering breakthrough that completely transforms the sector. Robotic-assisted surgery utilizes the accuracy of machines in conjunction with the proficiency of doctors, providing a less intrusive method for a broad spectrum of surgical procedures [14]. The da Vinci Surgical System, a cutting-edge robotic platform, enables surgeons to manipulate robotic arms with exceptional accuracy, converting their manual gestures into reduced-scale, meticulously regulated maneuvers inside the patient's body. Such high accuracy is especially advantageous in intricate procedures, such as prostatectomies and cardiac surgery [15]. Robotic systems provide an enhanced perception of spatial dimensions, enabling more precise dissection and suturing techniques. The influence of robotic surgery on surgical safety is complex and has multiple aspects. Automated operations are characterized by their minimally invasive approach, which typically involves smaller incisions [16]. This approach has several benefits, including decreased blood loss, reduced risk of infection, and quicker recovery times for patients. Surgeons can maneuver through anatomical structures more accurately, reducing harm to adjacent tissues. In addition, the robotic interface effectively filters and stabilizes hand movements, eliminating potential tremors and significantly improving the overall stability of surgical techniques [17]. Consequently, using robotics in surgical procedures has led to substantially decreased problems, enhancing safety and efficiency.

Role of AI in Ensuring Safety During Surgical Procedures

The healthcare field has been significantly impacted by the emergence of AI, which can revolutionize various aspects such as diagnostics and tailored therapy [18]. AI plays a role in multiple aspects of surgical safety, encompassing preoperative planning, intraoperative decision-making, and postoperative care. Before surgery, AI algorithms examine patient data, including medical records, imaging scans, and genetic information, to evaluate the person's risk profile and customize the surgical strategy accordingly [19]. This customized risk evaluation enables surgeons to take preemptive measures to address probable issues, maximizing patient outcomes. During surgery, AI is involved in identifying and examining images. Computer vision algorithms can analyze surgical photos in real time, accurately recognizing anatomical features, emphasizing any irregularities, and offering decision-making assistance to the surgical team [20]. This not only improves the surgeon's understanding of the situation but also aids in promptly identifying and preventing problems. AI-driven robotic systems can quickly change their movements in response to unexpected obstacles encountered during surgery, adapting to the changing surgical environment.

After surgery, AI plays a role in monitoring and forecasting patient outcomes. Machine learning algorithms examine postoperative data, including vital signs, laboratory findings, and recovery status, to detect patterns that suggest possible problems [21]. Early detection enables timely management, mitigating negative occurrences and enhancing postoperative care. Although incorporating AI into surgical safety shows excellent potential, it is crucial to carefully address ethical concerns, data protection, and the ongoing validation of algorithms [22]. Ensuring patient safety and upholding moral norms are essential to adequately developing and implementing AI technology in surgery.

State-of-the-Art Imaging Techniques

Sophisticated imaging tools have revolutionized the process by which surgeons strategize and carry out medical treatments, offering intricate anatomical data that was previously inaccessible. MRI, CT, and other imaging techniques provide detailed, three-dimensional images of the patient's anatomy, allowing surgeons to observe features with exceptional clarity [23]. Before surgery, these imaging modalities assist in surgical preparation by thoroughly comprehending the patient's anatomy and the precise positioning of vital components. Surgeons can recognize probable difficulties and complexities, enabling them to engage in careful and detailed preoperative planning [24]. Real-time imaging techniques, such as intraoperative MRI and fluoroscopy, provide continuous input to guide the surgeon's operations and monitor the procedure's progress throughout surgery.

The advantages of improved imaging go beyond only visualizing. Surgical navigation systems that incorporate imaging data offer surgeons a detailed guide, improving accuracy in intricate surgeries. In neurosurgery, neuronavigation devices employ preoperative imaging to guide the surgeon precisely to specific targets with millimeter accuracy, which is of utmost importance [25]. Advanced imaging techniques undeniably enhance surgical safety by improving precision and minimizing the probability of mistakes. However, there are still obstacles to overcome. These factors encompass the expenses associated with equipment, the requirement for specialized training, and apprehensions about radiation exposure in specific imaging techniques [26]. To overcome these problems, adopting a well-balanced strategy that considers the advantages compared to the possible hazards is necessary. This will guarantee that incorporating sophisticated imaging techniques improves the overall safety of surgical procedures.

Factors about human behavior and psychology

The presence of humans is a fundamental and powerful aspect of surgical safety, as it affects the interactions and dynamics in the operating room and patient results [27]. Human factors comprise a wide range of parts, such as cognitive, social, and organizational elements, that contribute to the overall performance of the surgical team. It is crucial to acknowledge and comprehend these aspects to establish an environment that places patient safety as a top priority [27].

Psychological Distress and Physical Exhaustion

The operating room is a high-pressure workplace where stress and exhaustion are widespread concerns. Surgeons and the surgical team frequently encounter intricate procedures requiring prolonged concentration and attentiveness. Extended surgical procedures, unforeseen crises, and uncertain complications can lead to mental and physical exhaustion, jeopardizing decision-making and effectiveness [28]. To manage stress and exhaustion effectively, it is essential to implement methods such as taking regular breaks, ensuring sufficient rest periods between shifts, and fostering a supportive corporate culture that places a high value on the well-being of surgical teams [29].

Perception of the Surrounding Environment

Ensuring situational awareness is vital to surgical safety. Surgeons are required to handle a substantial volume of information, encompassing patient data and immediate feedback received during surgical procedures, as well as factors such as distractions, interruptions, or inadequate communication [30].

Surgical checklists and protocols

Surgical checklists and defined protocols have become essential to ensure patient safety in the operating room. The WHO Surgical Safety Checklist, implemented in 2009, has gained international recognition and widespread adoption as a standard [13]. The checklist comprises pre-anesthesia induction, pre-skin incision, and pre-patient departure from the operating theater. It is a methodical way to ensure that essential safety precautions are consistently followed during the surgical procedure [15].

Extensive research continually confirms the efficacy of surgical checklists in avoiding errors and improving patient safety. A groundbreaking study published in the New England Journal of Medicine showed that adopting the WHO Surgical Safety Checklist led to a substantial decrease in complications and death rates in various surgical procedures and environments [18]. The checklist functions as a cognitive tool, reminding team members to verify crucial actions, such as patient identification, site marking, antibiotic administration, and equipment preparedness.

Achievements and Results

Multiple instances of success highlight the significant influence of surgical checklists on patient outcomes. For example, when the list was introduced at the Ateneo de Manila University School of Medicine in the Philippines, it led to a 50% decrease in complications and a significant decrease in death rates [19]. Similarly, implementing surgical checklists by the National Health Service in the United Kingdom resulted in a significant 16% decrease in postoperative problems.

Surgical checklists decrease adverse occurrences and promote a culture of communication and collaboration among surgical teams. The list functions as a standardized framework that promotes transparent communication and enables all team members to express any issues or observations, irrespective of their hierarchical position [20]. The transition toward a more inclusive and communicative atmosphere aids in detecting and reducing potential faults.

To summarize, surgical checklists and standardized protocols, such as the WHO Surgical Safety Checklist, have demonstrated their efficacy as potent instruments in mistake prevention, complication reduction, and overall enhancement of patient safety [13]. Their success stems from their capacity to facilitate crucial actions and cultivate a culture of collaboration and communication that is vital in the intricate and high-pressure setting of the operating room [14].

Surgical Team Simulation Training

Simulation training has become a fundamental aspect of improving the abilities and readiness of surgical teams. Surgical procedures' ever-changing and uncertain characteristics require a learning environment that enables practitioners to refine their abilities, experiment with novel methods, and handle intricate situations within a regulated context [22]. Simulation training serves as a means to connect theoretical information with practical implementation, offering a secure environment for experiential learning while ensuring the safety of patients.

Elements of Simulation Training

Simulation training spans many methods, from essential task trainers to advanced, very realistic simulators that accurately recreate the complexities of medical procedures. Utilizing virtual reality and augmented reality technologies has enhanced simulation training by fully engaging surgeons in authentic scenarios [23]. These technologies allow medical professionals to conduct simulated procedures, encounter accurate anatomical differences, and engage with patient avatars that resemble real-life individuals.

The simulation scenarios aim to recreate ordinary and high-risk situations accurately, enabling surgical teams to rehearse and enhance their reactions to unforeseen circumstances [24]. This includes the resolution of equipment malfunctions, the effective handling of unexpected issues, and the enhancement of communication and collaboration among team members. The debriefing process that follows simulation sessions is of equal importance since it allows for reflection, feedback, and ongoing enhancement [25].

Improving Team Dynamics

Simulation training has a significant advantage in improving teamwork dynamics in the operating room. Surgical operations necessitate inherent collaboration, demanding flawless communication and coordination among team members [26]. Simulation scenarios frequently incorporate complete surgical teams comprising surgeons, nurses, anesthesiologists, and additional supporting personnel to cultivate an authentic portrayal of the collaborative aspect of surgical procedures.

The TeamSTEPPS program, created by the Agency for Healthcare Research and Quality, demonstrates the incorporation of simulation in team training. This curriculum focuses on fundamental cooperation abilities such as effective communication, situational awareness, mutual assistance, and leadership [26]. Through participation in simulated scenarios, surgical teams can hone these abilities in a safe environment, ultimately using them to enhance their performance in natural clinical settings.

Effect on Patient Safety

Simulation training enhances patient safety by addressing critical factors that affect surgical results. Firstly, it enables practitioners to improve their technical proficiency, encompassing fundamental procedures and intricate surgeries, decreasing the probability of errors during surgical procedures [27]. Furthermore, simulation promotes a proactive stance toward managing complications, guaranteeing that surgical teams are adequately equipped to handle unforeseen difficulties. Moreover, simulation training strengthens the collaborative culture within surgical teams by emphasizing communication and teamwork, reducing the likelihood of miscommunication and oversights. Multiple studies have shown evidence of the beneficial effects of simulation training on patient outcomes [28]. A meta-analysis published in the Journal of the American Medical Association revealed that simulation-based activity was linked to enhanced procedural skills and decreased patient problems [28]. Moreover, simulation training has proven advantageous in high-risk fields, such as heart surgery and neurosurgery, where accuracy and efficient collaboration are paramount.

Obstacles and Prospects

Although simulation training offers many benefits, its wider adoption encounters obstacles. These factors encompass financial limitations, restricted availability of high-fidelity simulators, and the necessity for devoted training time amidst demanding clinical schedules [29]. To surmount these obstacles, healthcare establishments, educators, and lawmakers must make a concerted endeavor to allocate resources toward simulation infrastructure, incorporate simulation into structured training programs, and acknowledge its enduring advantages for patient welfare. Simulation training is at the forefront of innovative methods to improve the skills and preparedness of surgical teams [29]. Simulation enhances technical proficiency, proactive complication management, and efficient teamwork, all crucial for guaranteeing patient safety in the intricate and ever-changing field of surgery [30].

Patient-centered approaches

Importance of Patient Engagement

Patient-centered care has become increasingly important in ensuring surgical safety. Acknowledging patients as engaged contributors in their healthcare journey is not solely an ethical concern but also a strategic method to enhance safety outcomes [31]. Engaging patients in their surgical treatment promotes empowerment, facilitates well-informed decision-making, and improves overall satisfaction. Furthermore, actively involved and well-informed patients are more inclined to comply with preoperative guidelines, adhere to postoperative care protocols, and promptly communicate any worrisome symptoms [32]. As a result, they play a vital role in reducing problems.

Approaches for Educating and Involving Patients

Patient education is a crucial aspect of surgical care that focuses on the needs and preferences of the patient. It is essential to offer clear and easily understandable information regarding the intended procedure, risks, and expectations after the surgery. Employing multimedia tools, such as movies and brochures, helps augment the comprehension of intricate medical topics [32]. Facilitating patient involvement in shared decision-making, wherein they actively contribute to conversations regarding treatment alternatives and their consequences, guarantees that their values and preferences are considered during the decision-making process [33]. In addition, patient advocates or navigators can provide supplementary assistance by addressing issues and enhancing communication between patients and healthcare providers [34]. In the end, providing patients with knowledge and involving them actively in their treatment enhances the safety of surgical procedures and fosters a cooperative and trustworthy partnership between healthcare professionals and patients.

Efforts to enhance the overall quality of a process or system

Exemplary Cases of Enhancing Quality

Implementing quality improvement initiatives is now essential for enhancing surgical safety standards. Various instances of success exemplify the influence of these activities. The Michigan Surgical Quality Collaborative (MSQC) successfully implemented a data-driven quality improvement program, resulting in a substantial decrease in complications and notable enhancements in outcomes at the hospitals involved [35]. Through the systematic collection and analysis of data on surgical outcomes, MSQC has discovered optimal methods and areas that need improvement, resulting in a tangible increase in patient safety [36]. Moreover, the implementation of surgical safety checklists, influenced by the WHO Surgical Safety Checklist, has gained international recognition as a comprehensive effort to establish uniform safety protocols and minimize mistakes in various healthcare environments.

Effects of Data Analysis and Ongoing Monitoring

Data analysis and ongoing monitoring are crucial in quality improvement programs. Healthcare facilities gather and examine surgical outcome data to discern patterns, trends, and areas needing enhancement. Continuous monitoring allows for immediate feedback, enabling timely intervention and correction of practices [37]. Surgical teams and administrators can utilize dashboards that present critical performance data to monitor progress and detect anomalies. Incorporating electronic health records enhances the efficiency of data management, facilitating effortless retrieval of patient information and evidence-based decision-making [38]. Employing predictive analytics, machine learning, and AI can augment the predictive capacity of data analysis, enabling the detection of possible dangers before they increase. Continuous monitoring of surgical safety through comprehensive quality improvement activities ensures that interventions stay responsive to developing issues. Healthcare facilities can enhance surgical safety and decrease adverse occurrences by cultivating a culture that promotes ongoing learning and improvement [39].

Advancements in surgical instrumentation

Recent Innovations in Surgical Instruments

Surgical treatments have greatly benefited from advancements in surgical instrumentation, which have enhanced precision and safety. Recent innovations span various technologies and materials to improve surgeons' abilities and reduce dangers [40]. Robotics, for example, has transitioned from being a novelty to a widely used tool in surgery. Robotic-assisted surgery offers doctors improved manual control, a three-dimensional view, and the capability to perform minimally invasive treatments with unparalleled accuracy [41]. This decreases the extent of procedures and lessens harm to nearby tissues, resulting in quicker recovery times and fewer complications.

Moreover, incorporating AI into surgical instruments has brought forth functionalities such as picture identification, immediate decision assistance, and prognostic analysis. AI algorithms can analyze extensive datasets, assisting surgeons in preoperative planning, risk assessment, and making decisions during surgery. Intraoperative MRI and CT are advanced imaging tools that enhance surgical instrumentation by offering precise anatomical information, enabling accurate navigation and focused interventions [42]. Advancements in instrumentation have made significant contributions to enhancing safety. The safety benefits of novel instrumentation are diverse and complex. The utilization of robotic systems and AI-powered equipment in surgical procedures significantly reduces the possibility of errors due to their high level of precision. Surgeons can maneuver intricate anatomical structures with enhanced precision, hence minimizing the likelihood of unintended harm [43]. Advanced devices with minimally invasive characteristics yield smaller incisions, resulting in less blood loss, lower infection rates, and accelerated patient recovery. Moreover, instrument advancements play a role in establishing uniformity in surgical procedures. Advanced instrumentation enables standardized protocols and procedures, ensuring consistent care delivery among surgical teams and institutions [44]. The standardization process and continuous training and instruction on emerging technology promote the overall safety and quality of surgical procedures.

To summarize, advancements in surgical tools, such as robotics, AI, and enhanced imaging methods, are profoundly impacting the area of surgery [45]. These innovations enhance the accuracy and effectiveness of surgical treatments while significantly improving patient safety through risk reduction, complication reduction, and the promotion of standardized surgical care.

International viewpoints on the safety of surgical procedures

Analyzing and Comparing the Safety Practices in Surgery

Implementing surgical safety practices varies significantly worldwide since cultural, institutional, and economic considerations impact it. Although certain fundamental principles are universally applicable, such as infection control and patient consent, the execution and emphasis on specific safety measures can vary considerably [46]. High-income countries with robust healthcare systems typically implement standardized protocols, innovative technologies, and strict regulatory frameworks to enhance surgical safety practices [46]. Conversely, low- and middle-income nations may encounter obstacles such as scarce resources, insufficient infrastructure, and disparities in cultural perspectives on healthcare.

Impact of Culture and Systems on Safety Measures

Cultural and systemic factors significantly impact the development and implementation of surgical safety protocols. Cultural perspectives on healthcare, encompassing patient self-governance, well-informed agreement, and the divulgence of medical data, can impact the interaction dynamics between healthcare professionals and patients. Systemic elements, such as the allocation of funds for healthcare, the availability of technology, and regulatory structures, additionally influence the differences in safety protocols [47]. For instance, adopting standardized safety measures may be more feasible in nations with centralized healthcare systems than in countries with decentralized or privatized healthcare frameworks. Obstacles to establishing consistent surgical safety regulations across different settings encompass discrepancies in education and training, technological availability inequalities, and healthcare administration inconsistencies [48]. To address these disparities, engaging in cooperative endeavors, establishing multinational alliances, and acknowledging that ensuring surgical safety is a collective obligation that extends across the globe are necessary.

## Conclusions

Patient-centered methods, quality improvement initiatives, innovations in surgical instrumentation, and global viewpoints influence the development of surgical safety. Understanding the need for patient involvement, establishing vital quality improvement programs, adopting advanced technologies, and recognizing the cultural and systemic complexities of surgical safety practices all contribute to a complete and ever-changing framework. As we progress in ensuring surgical safety in the future, incorporating these varied components will play a crucial role in attaining the best possible results for patients across the globe. Pursuing safer surgeries necessitates a dedication to ongoing education, a proactive stance on enhancing quality, and the cooperative endeavors of healthcare practitioners, policymakers, and patients. In essence, the quest for surgical safety is an international effort that goes beyond national boundaries, highlighting the common objective of providing excellent, patient-focused healthcare in the continuously evolving surgery domain.
